# Improved pharmacokinetic and pharmacodynamic profile of a novel PEGylated native *Erwinia chrysanthemi* L-Asparaginase

**DOI:** 10.1007/s10637-021-01173-8

**Published:** 2021-09-01

**Authors:** Tapasvi Modi, David Gervais

**Affiliations:** Porton Biopharma Limited, Porton Down, Salisbury, Wiltshire, SP4 0JG UK

**Keywords:** L-Asparaginase, Leukaemia, ALL, *Erwinia chrysanthemi*, *Escherichia coli*, PEG, Pharmacokinetic, Pharmacodynamic, Therapeutic enzyme, Half-life

## Abstract

*Introduction*. Erwinase® (native *Erwinia chrysanthemi* L-Asparaginase (nErA)) is an approved second-line treatment for acute lymphoblastic leukaemia (ALL) in children and adolescents, who develop hypersensitivity or neutralising antibodies to *E.coli* derived L-Asparaginases (ASNases). However, nErA has a short in vivo half-life requiring frequent dosing schedules in patients. In this study, nErA was covalently conjugated to PEG molecules with the aim of extending its half-life in vivo. *Methods*. Firstly, efficacy of this novel product PEG-nErA was investigated on human ALL cell lines (Jurkat, CCRF-CEM and CCRF-HSB2), in vitro. Secondly, its pharmacokinetic (PK) and pharmacodynamic (PD) characteristics were determined, in vivo (12 rats in each group). *Results*. It was found that the specific activity (U/mg of enzyme) and the kinetic constant (K_M_) of nErA remained unaltered post PEGylation. PEG-nErA was shown to have similar cytotoxicity to nErA (IC_50_: 0.06–0.17 U/mL) on human ALL cell lines, in vitro*.* Further, when compared to nErA, PEG-nErA showed a significantly improved half-life in vivo, which meant that L-Asparagine (Asn) levels in plasma remained depleted for up to 25 days with a four-fold lower dose (100 U/kg) compared with 72 h for nErA at 400 U/kg dose. *Conclusion*. Overall, this next generation product PEG-nErA (with improved PK and PD characteristics compared to nErA) would bring a significant advantage to the therapeutic needs of ALL patients and should be further explored in clinical trials.

## Introduction

L-asparaginase (ASNase) is an integral part of the treatment of acute lymphoblastic leukaemia (ALL) in children and adolescents. ASNase catalyses the production of L-Aspartic acid (Asp) from L-Asparagine (Asn). ALL cells lack Asn synthase activity [1, 2], which means that ALL cells cannot generate their own supply of Asn and depend on circulating endogenous Asn. The mechanism of action of ASNase treatment is to deplete circulating Asn in blood, starving ALL cells of the essential amino acid Asn leading to cell death. There are now four ASNase preparations that have been approved for ALL treatment. For years, options for ASNase treatment have included native *Escherichia coli* ASNase (nEcA), native *Erwinia chrysanthemi* ASNase (nErA) and PEGylated *Escherichia coli* ASNase (PEG-nEcA). Recently, a recombinant version of *Erwinia chrysanthemi* ASNase has been approved by the US FDA [3]. However, to date no PEGylated *Erwinia*-based ASNase has been approved for clinical use in ALL treatment.

Most ALL patients receive PEG-nEcA as a first-line treatment, however around 30–40% of those patients develop hypersensitivity reactions [4, 5]. This hypersensitivity can either be due to an adverse immune reaction to the enzyme or due to the generation of neutralising antibodies (often referred to as silent inactivation), resulting in reduced clinical function of the enzyme [6]. nErA is immunologically distinct from PEG-nEcA and does not present cross-reactivity to antibodies generated from PEG-nEcA [7]. Hence, nErA (or, as of July 2021, rErA) is offered as a second-line treatment to those ALL patients who develop hypersensitivity to PEG-nEcA. Erwinase® or Erwinaze® are the proprietary names for nErA*.* Erwinase® was first developed in the late 1960s and early 1970s at Porton Down, UK [8, 9] and is marketed in many countries throughout the world.

Both nEcA and nErA consist of a homotetramer of 35 kDa subunits and although they are structurally similar proteins, their sequences have only 46% identity [10]. Both Asn and L-Glutamine (Gln) act as substates for nEcA and nErA enzymes, however they have significantly different affinities, as determined by the Michaelis constants (K_M_) [11, 12]. Similar to that of nEcA, the in vivo half-life of Erwinase® is low in comparison to PEG-nEcA, resulting in the requirement for frequent dosing of the native enzymes (several times per week) compared with the PEGylated version of nEcA.

It follows that one potential way to increase nErA half-life and reduce the dosing frequency is to PEGylate the enzyme, in a manner analogous to that of PEG-nEcA. Understanding the impact of PEGylation on the in vivo half-life of the drug is a critical step in the development of this form of nErA. Thus, in the research described here, PEGylation of nErA was investigated both in vitro on human ALL cell lines and in vivo in terms of its pharmacokinetic (PK) and pharmacodynamic (PD) profile.

## Methods and materials

All reagents were purchased from Sigma Aldrich, UK, unless specified otherwise.

### PEGylation of nErA

nErA drug substance (DS) was obtained from Porton Biopharma Limited (PBL), Porton Down, UK, manufactured as described previously [13]. nErA DS was then conjugated to NHS-activated monomethoxy poly (ethylene glycol) (PEG or mPEG) with a mean molecular weight of 5 kDa with succinimidyl carbonate (SC) linkers (Laysan Bio, USA), using standard protocols [17].

### Specific activity and K_M_ measurement

The specific activity (U per mg of enzyme) and K_M_ of nErA and PEG-nErA were determined using methods described previously [14].

### In vitro Study—Cytotoxicity assay

The in vitro efficacy of PEG-nErA vs nErA was assessed using cytotoxicity assay at an external contract research organisation (CRO) (Cyprotex, UK). The enzymes (± PEG) were supplied by PBL and the cell lines were acquired from ATCC, UK. The following acute lymphoblastic leukaemia (ALL) cell lines were used: Jurkat, CCRF-CEM and CCRF-HSB2. Cell lines used for negative control included non-ALL cell lines 1301 and THP1. Briefly, the cell lines were cultured in RPMI 1640 medium supplemented with 200 mM Glu, 10% Foetal bovine serum and 1% Penicillin–Streptomycin. nErA and PEG-nErA samples diluted in water were added to wells of a 96-well plate and serially diluted to arrive at final concentrations between 50U/mL and 5 × 10^–5^ U/mL, with one set of control wells (water only) in triplicates. To each well, 100µL of cells was added at either 1 × 10^5^ or 2 × 10^5^ cells per 100µL. The plates were incubated at 37 °C for 72 h. Then, 20µL of MTT (3-(4,5-Dimethylthiazol-2-yl)-2,5-Diphenyltetrazolium Bromide) (5 mg/mL) reagent was added to each well and the plate incubated for a further 2 h at 37 °C. Finally, 100µL of solubilisation reagent (0.37% hydrochloric acid in isopropanol) was added and mixed thoroughly with a pipette before reading the plate absorbance at 570 nm with background at 750 nm using a plate reader (Molecular Devices, UK). The cell viability data was normalised and IC_50_ data were measured at 72 h using Graph-Pad Prism 7.

### In vivo study

The in vivo efficacy of PEG-nErA versus an nErA control was assessed at an external CRO (Envigo, UK). Briefly, male CD Sprague–Dawley rats (Charles River, UK) received a single intravenous administration of control PBS (0 U/kg), nErA (400 U/kg), or PEG-nErA (1, 5, 25, 100 or 400 U/kg) (Table 1). There were 12 animals in each group. Venous blood samples were taken from each animal at defined timepoints (pre-dose and up to 1032 h) for measuring ASNase activity (PK parameter) and Asn and Gln concentration (PD parameter). Blood samples were taken in K_2_EDTA tubes and separated by centrifugation (2000 g at 4 °C for 10 min) before aliquoting the resulting plasma into 2 separate vials and freezing at -70 °C. Samples were shipped to PBL on dry ice. The animal experiment protocol was approved by the local ethics committee at Envigo.

**Table 1:** Number of rats in the in vivo study. Animals were of uniform age and animal weights were 336-405 g before dose administration

**Study Group**	**Number and sex of animals**	**Dose route**	**Dose level (U/kg)**	**Treatment**
1	12 M	IV	0	Vehicle
2	12 M	IV	400	nErA
3	12 M	IV	1	PEG-nErA
4	12 M	IV	5	PEG-nErA
5	12 M	IV	25	PEG-nErA
6	12 M	IV	100	PEG-nErA
7	12 M	IV	400	PEG-nErA

### Measurement of plasma ASNase activity

ASNase activity in plasma samples was measured using the method described in Lanvers et al*.,* [15]. Briefly, AHA solution (10 mM L-Aspartic acid Beta-Hydroxamate (AHA) was prepared in 50 mM MOPS, 0.015% (w/v) BSA, pH 7.2) was incubated at 37 °C for at least 10 min. In a 96-well microplate, 10µL of plasma and 90µL of pre-warmed AHA solution was added. As a reference standard, 10µL of nErA reconstituted in water with known activity was substituted for plasma. The microplate was incubated at 37 °C for 40 min and 125µL of 24.5% (v/v) trichloroacetic acid was added. The plate was centrifuged at 2500 rpm for 5 min. In a separate microplate, 150µL of 1 M disodium carbonate and 50µL of 2% 8-hydroxyquinoline in ethanol were added to each well. Then, 50µL of the supernatant from the original plate was added, and the plate was heated at 55 °C for 40 min, allowed to cool to room temperature before reading in a plate reader (Molecular Devices, UK) at 710 nm.

### Measurement of plasma Asn and Gln levels

Plasma Asn and Glu concentrations were determined using LC–MS/MS at Q3 Analytical Ltd. Briefly, Asn and Glu were weighed, dissolved in deionised water to a concentration of 1 mg/mL. Midodrine was dissolved in dimethyl sulphoxide (DMSO) as a 1 mg/mL stock solution and diluted to 1 μg/mL in acetonitrile to make the internal standard (IS) solution. Samples were prepared by mixing 50μL of plasma (standard or sample) with 150μL of IS solution, centrifuged and the supernatant transferred to a 1.5 mL Eppendorf. The solution was reduced to 50µL using a centrifugal evaporator (Genevac, UK) for 45 min at 40 °C and added to 70μL AccQ Tag borate buffer (Waters, UK) and 20μL Waters AccQ Tag reagent solution (Waters, UK) (1 vial dissolved in 4 mL reconstitution solution). The tube was sealed and incubated at 60 °C for 10 min. After cooling to room temperature, 5μL was injected onto the LC–MS system. Amino acids were separated on a reverse-phase symmetry C_18_ column (Waters, UK), using gradient of mobile phase A (0.1% formic acid, 5 mM heptafluorobutyric acid) and mobile phase B (0.1% formic acid in acetonitrile, 5 mM heptafluorobutyric acid) for 7 min. The peak area ratios of the amino acids to the IS were used as a surrogate measure of the concentrations of each amino acid.

### Statistics

For cytotoxicity assays, IC_50_ values were obtained using GraphPad Prism 7 and are presented with the standard error from four-parameter curve fitting. The difference in efficacy of nErA vs PEG-nErA in vitro was calculated using t-test, p value of < 0.05 was considered statistically significant. For PK/PD studies, results were presented as ± 1 standard deviation around the mean.

## Results

### PEGylation of nErA

PEGylation of nErA (up to 350 mol PEG per mole 35kD monomer) did not alter the specific activity and K_M_ values of nErA (Table 2). Using a capillary isoelectric focussing technique, it was previously found that PEGylation of nErA incorporated approximately 7 mPEG molecules per 35 kDa nErA monomer for reaction molar ratios on the order of 40 PEG molecules to one enzyme molecule [16]. PEG-nErA was formulated using trehalose and lyophilised, which was found to offer extended stability at 5 °C ± 3 °C for up to 3 years (data not shown).

**Table 2:** Specific activity and K_M_ of nErA and PEG-nErA

	**nErA**	**PEG-nErA**
Specific Activity	1170 ± 41	1166 ± 71
K_m_ (µM)	30.4 ± 2.5	32.0 ± 4.1

### Cytotoxicity of PEG-nErA vs nErA, in vitro

**Fig. 1:**
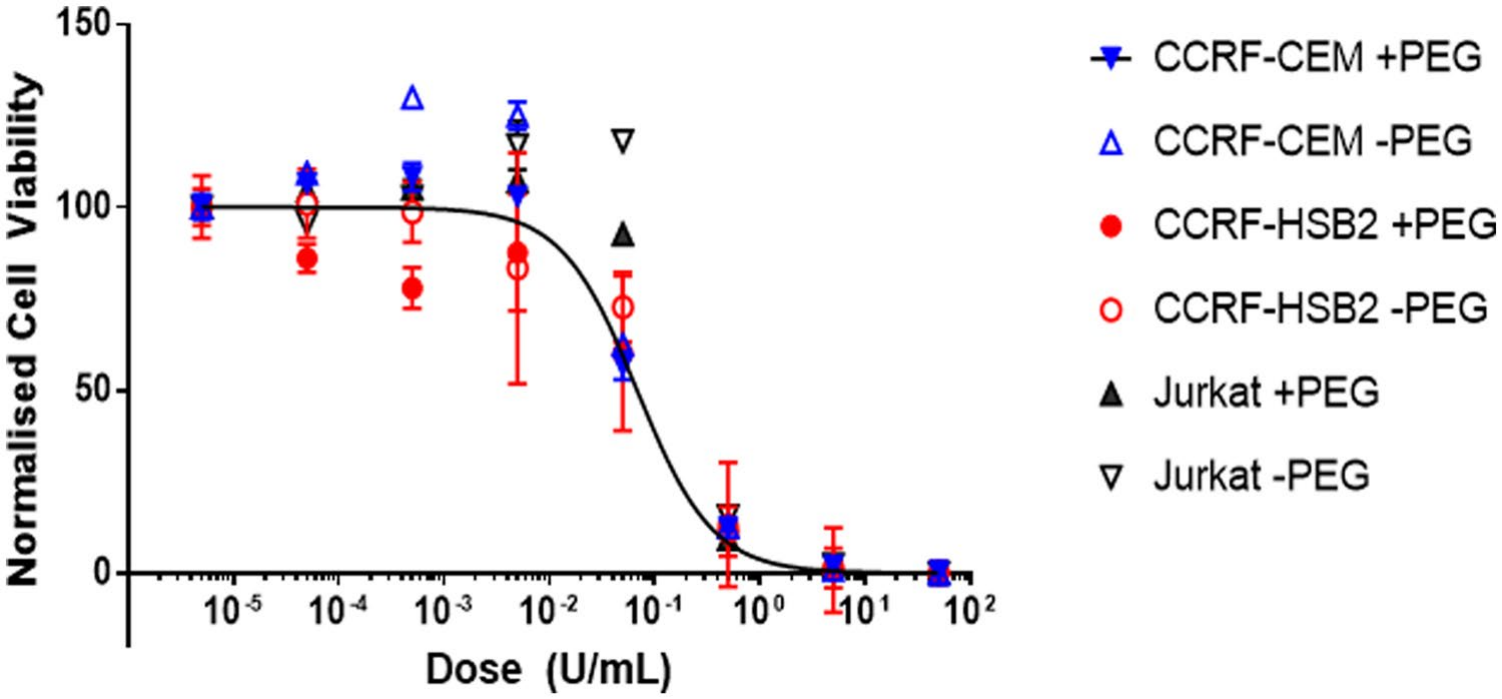
Cytotoxic effect of PEG-nErA and nErA on human ALL cell lines after 72 h, in vitro using MTT assay. Cells were treated with increasing dose ± PEG Erwinase (5 × 10^–5^ to 50 U/mL), in triplicates. The data shows that PEGylation of nErA did not alter the overall cytotoxicity of nErA against human ALL cells. IC_50_ values were obtained using GraphPad Prism and are presented with the standard error from 4-parameter curve fitting

The viability of ALL cell lines treated with serial dilutions of nErA and PEG-nErA was measured at 72 h using the MTT assay. The data showed similar ALL cell viability when cell lines were treated with nErA or PEG-nErA (Fig. 1). The absolute viability data differed from cell line to cell line and this may be due to simple variation in the way the various cells reacted with the MTT reagent. IC_50_ values, representing the interpolated enzyme dose at which 50% of the ALL cells were killed were calculated. Overall, both nErA and PEG-nErA showed equivalent cytotoxicity on human ALL cell lines after 72 h (Fig. 1, Table 3). Note that cytotoxicity data in Fig. 1 were normalised using Graph Pad Prism 7 so that all data sets were on a 0 – 100% scale (0% representing the lower horizontal asymptote and 100% representing the upper horizontal asymptote), in order to allow a direct comparison between all data sets. In the negative control cell lines, there was no cytotoxic effect from both nErA and PEG-nErA (data not shown).

**Table 3:** Cytotoxicity IC_50_ data (72 h Incubation) for nErA and PEG-nErA. The IC_50_ value represents the interpolated enzyme dose at which 50% of the ALL cells were killed. IC_50_ values were obtained using GraphPad Prism 7 and are presented with the standard error from four-parameter curve fitting. Note that the GraphPad software was not able to resolve the standard deviation of the Jurkat (nErA control arm)

**Cell Line**	**ASNase**	**IC**_**50**_ **(U/mL of Enzyme)**
CCRF-CEM	nErA	0.07 ± 0.04
CCRF-CEM	PEG-nErA	0.07 ± 0.01
CCRF-HSB2	nErA	0.10 ± 0.02
CCRF-HSB2	PEG-nErA	0.06 ± 0.03
Jurkat	nErA	0.40
Jurkat	PEG-nErA	0.17 ± 0.03

### In vivo studies

#### Effect of treatment on plasma Asn and Gln concentration in vivo

**Fig. 2:**
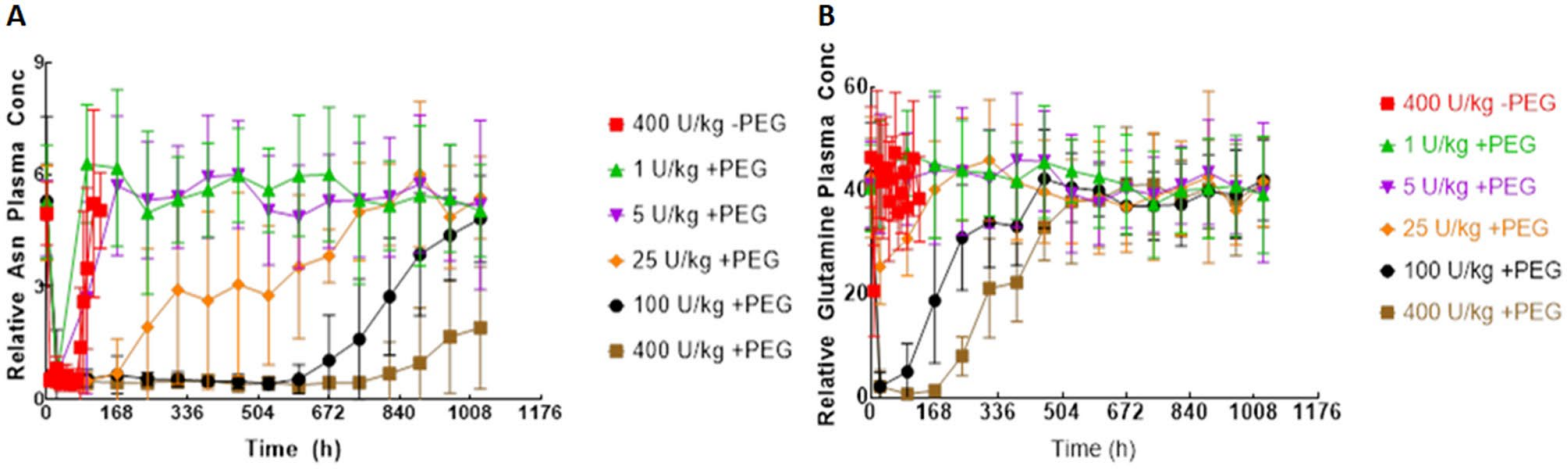
Amino Acid Profiles from in vivo Study, Fig. 2**A**: L-Asparagine levels, Fig. 2**B**: L-Glutamine levels. Each data point represents the mean of results from 12 animals. Error bars refer to ± 1 standard deviation around the mean

The effect of PEGylation of nErA on plasma Asn and Gln was clearly observed during in vivo investigation. With respect to Asn concentration in plasma, animals receiving 400 U/kg nErA (without PEG) showed a typical response in terms of the timing and duration of the Asn trough, where Asn is depleted after dosing (time ‘zero’) to a non-detectable level for approximately 72 h before rebound to endogenous pre-dose levels. However, for PEG-nErA, dependent on dose, animals maintained a much longer Asn trough period. In the groups receiving 1, 5, 25, 100 and 400 U/kg of PEG-nErA, Asn depletion followed a dose-dependent relationship (Fig. 2A). Plasma Asn levels remained at undetectable levels for at least 25 and 31 days at 100 and 400U/kg PEG-nErA dose levels, respectively. For all dose levels except the highest (400U/kg PEG-nErA), complete rebound in Asn levels was observed over the duration of the study; for the highest dose level, only a partial rebound was observed.

For plasma Gln, a reduction from endogenous levels was only observed for PEG-nErA. For the non-PEGylated nErA control, there was no detectable reduction in plasma Gln level after dosing. At PEG-nErA doses ≥ 25U/kg, plasma Gln levels were depleted in a dose-dependent manner (Fig. 2B). There was no detectable reduction in circulating Gln levels for the lowest two PEG-nErA groups (1 and 5U/kg).

### Plasma ASNase activity

**Fig. 3:**
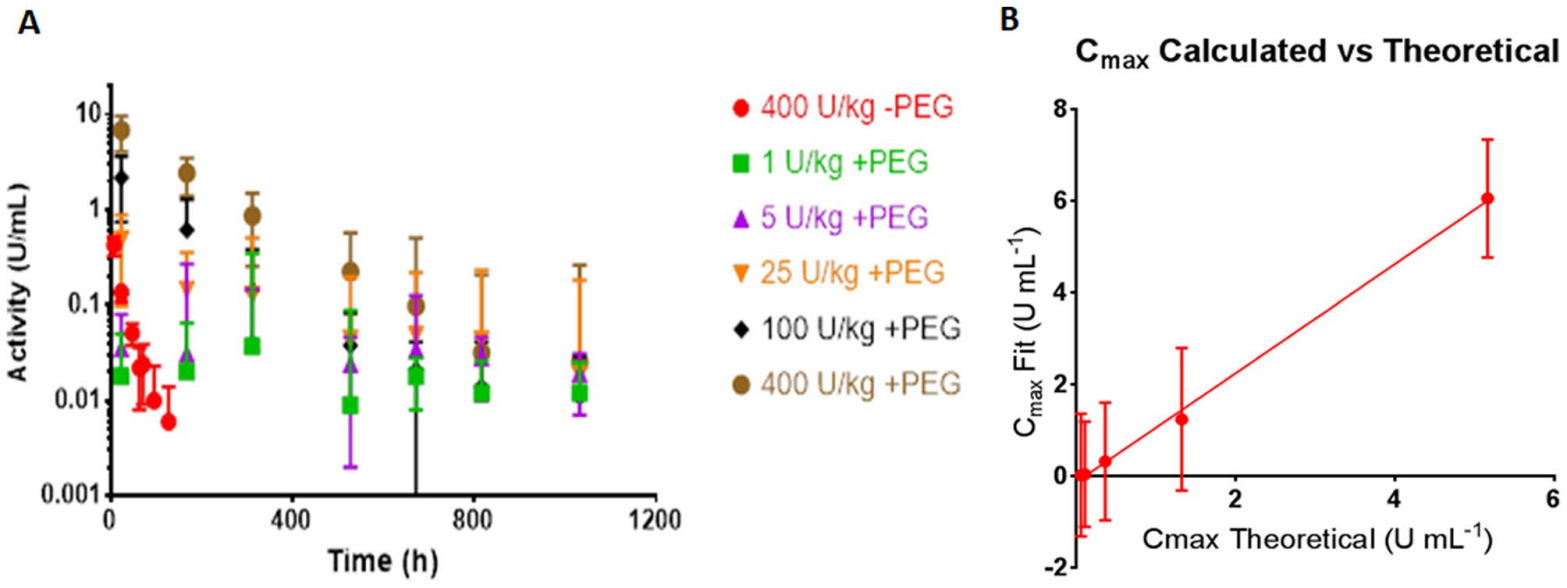
Pharmacokinetic study performed in rats (n = 12 per dose, per group). Figure 3**A**: L-Asparaginase activity measured in rat plasma, determined using enzymatic activity method described in Lanvers et al*.*. [15]. Each data point represents the geometric mean of results from 12 animals. *Error bars* refer to ± 1 standard deviation around the geometric mean. Figure 3**B**: Correlation between C_max_ from Regression Analysis and Theoretical C_max_. Error bars refer to ± 1 standard error around the fitted C_max_ data

nErA PEGylation was found to prolong the presence of ASNase in vivo in a dose-dependent manner. The plasma ASNase activity levels remained high in the PEG-nErA groups for longer duration as can be observed by the decreasing slope of the activity data points with increasing PEG-nErA dose (Fig. 3). Plasma activity was measured at 0.05 U/mL in the nErA group at 48 h, and a similar activity was measured at 32 days in the 400 U/kg PEG-nErA group. Data in Fig. 3 was plotted as the geometric mean of each activity from the 12 animals in a group. In lieu of a direct PK measurement, the plasma activity data in Fig. 3 were used as a surrogate marker to calculate PK parameters for this study.

The C_max_ from the activity data was estimated using a curve fitting technique (Table 4). As the animals in the study received only one dose of drug, by definition the C_max_ is the concentration of drug in the animal’s blood at time zero. However, no plasma samples were taken from the animals in the PEG-nErA groups until 72 h, so a regression was performed and the C_max_ back-calculated as the y-intercept. The geometric mean activity data were plotted per Fig. 3 and fitted using a logarithmic curve fit in Graph Pad Prism 7. Graph Pad was also used to calculate the area under the curve (AUC). In addition to C_max_ and AUC, a number of additional PK parameters including clearance and half-life were calculated using standard equations for a single intravenous bolus [13]. Note that the calculated PK parameters were not meaningful at the lower dose levels (1 and 5 U/kg), as the enzyme activity levels were low for these dose levels resulting in poor curve fitting. Moreover, this study was geared towards observing the effects of higher PEG-nErA doses, and hence the time spacing between observations and samples was insufficient to observe effects at the lowest PEG doses. Hence, PK parameters for the 1 and 5 U/kg dose levels are not reported in Table 4.

**Table 4:** Pharmacokinetic (PK) and Pharmacodynamic (PD) Parameters were calculated from activity data in Fig. 3. The C_max_ (column 2) was estimated as the y-intercept from a logarithmic curve fit of the geometric mean of activity data. AUC was calculated using GraphPad Prism 7. Other parameters were then calculated using the intravenous bolus equations of [17]. The C_max_ theoretical was the mean activity actually delivered to each treatment group assuming a blood volume per animal of 28.5 mL in a 365 g rat

**Parameter**	**Area Under the Curve, AUC**	**Estimated Maximum Activity in vivo, Cmax**	**Half Life, t**_**1/2**_	**Cmax theoretical**	**Clearance, CL**
**Unit**	**U h mL**^**−1**^	**U mL**^**−1**^	**h**	**U mL**^**−1**^	**U h**^**−1**^
**25 U/kg + PEG**	104.3	0.3 ± 1.3	82.2	0.32	0.24
**100 U/kg + PEG**	283.8	1.2 ± 1.6	78.4	1.28	0.26
**400 U/kg + PEG**	1062.0	6.1 ± 1.3	96.5	5.12	0.17
**400U/kg -PEG**	8.2	0.3 ± 1.0	9.7	5.12	31.2

Observing the AUC data, a dose-dependent increase was observed for the PEGylated enzyme, and the effect of PEGylation on AUC was evident (comparing nErA and PEG-nErA groups at 400 U/kg dosage). A predictable increase in half-life (t_1/2_) was also observed, at 9.7 vs 96.5 for nErA and PEG-nErA groups respectively at the 400U/kg dosing level. Further as expected, the clearance rate was significantly higher for animals in the nErA group vs those receiving PEG-nErA.

The calculated C_max_ from logarithmic regression may also be compared to the theoretical C_max_. The theoretical C_max_ (also shown in Table 4) is calculated based simply on the amount of drug dosed to an animal divided by the animal’s blood volume. The average weight of a rat at time zero in this study was 365 g. The blood volume of a 365 g rat was estimated at 28.5 mL and used to calculate the theoretical C_max_. A plot of this theoretical C_max_ versus the C_max_ obtained from linear regression of the activity data (Fig. 3B) showed that a linear relationship exists between the two sets of data. Based on this, it can be concluded that the PK data obtained in this study are valid and represent a reasonable representation (within the constraints of using a surrogate PK marker) of the animal metabolism of PEG-nErA.

## Discussion

This study demonstrated that PEGylation of nErA had no impact on its specific activity and kinetic enzyme performance. PEGylation of biomolecules sometimes results in decreased drug-target binding affinity due to steric interference [18]. Similar studies have reported loss of 35–60% of the ASNase activity as a result of PEGylation [19, 20]. The apparent lack of impact of PEGylation may be due to the characteristics of the nErA molecule, but further investigation would be required to understand this relationship.

The in vitro cell viability data and IC_50_ values demonstrated that nErA and PEG-nErA appear identical with respect to cytotoxicity against human ALL cells. Furthermore, no cytotoxic effect was observed in the negative control cell lines (*i.e.,* non-ALL cell lines) for both nErA and PEG-nErA. This further demonstrated that PEGylation was not the pivotal variable in cytotoxicity, and that the relationship between nErA (or ASNase more generally) and the ASNS negative characteristic of ALL cell lines is the important determinant for cytotoxicity [12].

The in vivo study showed that PEG-nErA had improved PK and PD characteristics in comparison to nErA. PEG-nErA exhibited a significantly improved half-life and depleted plasma Asn levels for longer duration in a dose-dependent manner in rats, in comparison to nErA. A single intravenous dose of 400 U/kg was found to be sufficient to deplete the plasma Asn levels for 31 days with PEG-nErA vs 72 h with nErA. In fact, PEG-nErA at ≥ 25U/kg dose outperformed nErA in terms of duration of Asn depletion, suggesting that 25–100 U/kg PEG-ErA dose could fit the typical two to three-week therapeutic window typically observed for currently used PEGylated ASNase products. Note that this final assertion does not take into account the potential effects of differences in treatment species with respect to dosing.

Further, there was no measurable depression in circulating Gln levels for the nErA and low-dose PEG-nErA groups (1 and 5U/kg dosage). However, plasma Gln levels remained depleted for a longer duration in PEG-nErA treated groups (≥ 25U/kg dosage) in a dose-dependent manner. There is significant debate in the scientific literature about the importance of anti-Gln activity in ASNase treatment of ALL. While glutaminase activity of ASNase is sometimes cited as being responsible for ALL side effects and toxicity in humans [21, 22], other literature reports claim that glutaminase side activity is critical to ALL blast apoptosis and overall efficacy of treatment [23–25]. Although the role of glutaminase activity in the clinical efficacy of *Erwinia* asparaginases and in particular PEG-nErA is not clear, the relationship between PEGylation and measurable, prolonged Gln depletion may be an important factor in any resulting clinical trials and thus, should continue to be monitored.

The plasma activity data compared well against the amino acid profile data. Plasma activity levels remained elevated for a longer duration in the PEG-nErA groups (48 h vs 32 days at 400 U/kg dose), and plasma activity also followed a dose-dependent relationship. Plasma ASNase activity level in the range of 0.05–0.4 U/mL is reported to be necessary for anti-leukemic therapeutic activity in humans [1, 4, 15, 26–33]. Plasma enzyme activity threshold of 0.1 U/mL was reached within only 24 h with nErA (400 U/kg), compared to 15 days with a lower dose of 100 U/kg PEG-nErA. This data compared well with other PEG-ASNase products (100 U/kg), reporting 0.1 U/mL plasma enzyme activity levels at 15 days in rats [34]. Furthermore, at lower plasma activity threshold of 0.01 U/mL, PEG-nErA appears to perform better at the same dose level (100 U/kg); this threshold was reached at 34 days with PEG-nErA vs 21 and 26 days with oncaspar and MC0609, respectively [34].

ALL patients who develop hypersensitivity or neutralising antibodies to *E.coli* based ASNase products rely on Erwinase® (nErA) as a second line treatment. However, Erwinase® has a limited supply issue, often leading to patient prioritisation [35]. Supposedly, recently approved JZP-458 (rErA produced in *Pseudomonas fluorescens*) potentially helps fulfil this unmet need by providing additional asparaginase preparation for those patients [3], however the hypersensitivity rate from this product is not yet understood or reported. Furthermore, PEG-nErA would also help resolve Erwinase® shortage problem, as clinically, it may be expected that PEG-nErA can be given at lower dosage and less frequently to patients compared with nErA or rErA, while achieving the same desired therapeutic outcome and improved patient experience.

PEGylation of biomolecules is already a well-established technology, shown to have significant net beneficial effects for patients [36–39]. Based on the results reported here, it is reasonable to expect that PEG-nErA would have an overall similar clinical safety and immunogenicity profile to nErA. However, further research will be necessary to advance this potential product variant to clinical use. It is envisaged that a pre-clinical study will be necessary to investigate the toxicity profile and PK/PD characteristics of PEG-nErA in a bigger animal model such as beagle dog. It is known that up to 33% of patients who switch from *E.coli* based ASNase products to nErA after developing clinical hypersensitivity suffer from allergic reactions to nErA [4]. It is expected that PEGylation of nErA would potentially reduce immunogenicity of nErA as well as the formation of neutralising anti-ASNase antibodies. Furthermore, repeated administration of PEGylated therapeutics is known to induce formation of anti-PEG antibodies associated with hypersensitivity reactions and rapid clearance [40, 41]. Hence, it would be useful to monitor anti-ASNase antibody and anti-PEG antibody levels during future pre-clinical studies. However, it has to be noted that measuring anti-drug antibody levels in animals may be of limited benefit in predicting clinical consequences in humans [42]. Subsequently, a phase I clinical trial in humans can be proposed to investigate safety and effective dose range in healthy volunteers.

## Data Availability

Yes.

## Abbreviations

AHA: L-aspartic β-hydroxamate
ALL: Acute Lymphoblastic Leukaemia
Asn: L-Asparagine
ASNase: L-Asparaginase
AUC: Area Under the Curve
C_max_: Maximum Concentration
CRO: Contract Research Organisation
DS: Drug substance
Gln: L-Glutamine
Glu: Glutamic acid
IC_50_: Interpolated Dose Representing 50% of Cells Killed
IS: Internal Standard
K_M_: Michaelis constant
K_2_EDTA: Potassium Ethylene Diamine Tetraacetic Acid
LC–MS/MS: Tandem Mass Spectrometry
mPEG: Monomethoxy poly(ethylene glycol)
MTT: (3-(4,5-Dimethylthiazol-2-yl)-2,5-Diphenyltetrazolium Bromide
nEcA: Escherichia coli ASNase
nErA: Native Erwinia chrysanthemi ASNase
PBL: Porton Biopharma Limited
PBS: Phosphate Buffered Saline
PD: Pharmacodynamics
PEG: Poly(ethylene glycol)
PEG-nEcA: PEGylated Escherichia coli ASNase
PK: Pharmacokinetics
SC-mPEG: Succinimidyl Carbonate Monomethoxy Poly(ethylene glycol)
SD: Standard Deviation
U: Units (of Enzyme
